# Imaging Sample Acidification Triggered by Electrochemically Activated Polyaniline

**DOI:** 10.1021/acs.analchem.2c03409

**Published:** 2022-09-27

**Authors:** Fabian Steininger, Alexander Wiorek, Gaston A. Crespo, Klaus Koren, Maria Cuartero

**Affiliations:** § Aarhus University Centre for Water Technology, Department of Biology, Section for Microbiology, Aarhus University, 8000 Aarhus, Denmark; ‡ Department of Chemistry, School of Engineering Science in Chemistry, Biochemistry and Health, KTH Royal Institute of Technology, SE-100 44 Stockholm, Sweden

## Abstract

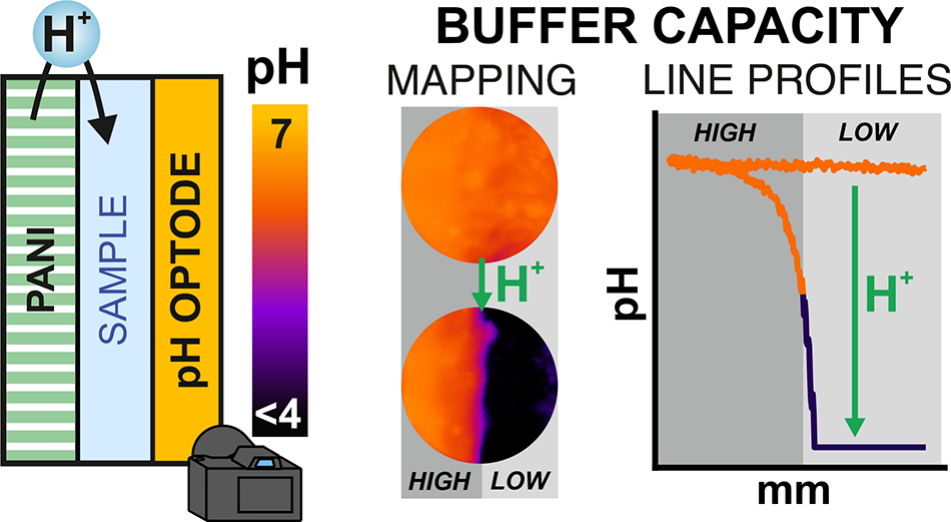

In this letter, we demonstrate 2D acidification of samples at environmental and physiological pH with an electrochemically activated polyaniline (PANI) mesh. A novel sensor–actuator concept is conceived for such a purpose. The sample is sandwiched between the PANI (actuator) and a planar pH optode (sensor) placed at a very close distance (∼0.50 mm). Upon application of a mild potential to the mesh, in contrast to previously reported acidification approaches, PANI releases a significant number of protons, causing an acid–base titration in the sample. This process is monitored in time and space by the pH optode, providing chemical imaging of the pH decrease along the dynamic titration via photographic acquisition. Acidification of samples at varying buffer capacity has been investigated: the higher the buffer capacity, the more time (and therefore proton charge) was needed to reach a pH of 4.5 or even lower. Also, the ability to map spatial differences in buffer capacity within a sample during the acid–base titration was unprecedentedly proven. The sensor–actuator concept could be used for monitoring certain analytes in samples that specifically require acidification pretreatment. Particularly, in combination with different optodes, dynamic mapping of concentration gradients will be accessible in complex environmental samples ranging from roots and sediments to bacterial aggregates.

Very often, the assessment of an analyte requires pretreatments based on the addition of reagents to the sample. This restricts the analysis operation to centralized laboratories and precludes point-of-care and on-site field measurements. While some attempts have been directed to combine and automate sampling and reagents’ addition in compact devices (e.g., paper strips for on-site alkalinity and phosphate detection),^1,2^ all-solid-state and/or reagentless approaches are desired to facilitate the final detection.

A common case of sample pretreatment is the change of its original pH. Effectively, disruptive approaches have emerged in recent years for successful pH modulation. For example, the pH of a solution can be locally adjusted via water electrolysis in a three-electrode cell: acidification/alkalinization occurs in the microenvironment of the counter and working electrodes. Early attempts (in the 1980s) for coulometric, reagentless titrations applied water splitting at gold electrodes and utilized sample confinement to decrease the analysis time to a matter of seconds while detecting the end point with potentiometry.^3,4^ However, this strategy is limited by possible side reactions in the sample due to the high overpotentials that are required.

Another option is the use of membranes enriched with protons in exchangeable positions, i.e., ion-exchange Donnan exclusion membranes.^5^ Despite being efficient, the membrane needs to be sandwiched between the sample and an acid that ensures the proton replenishment in the membrane. More recently, polyaniline (PANI) has been demonstrated as a material capable of releasing protons into confined water samples.^6^ When PANI is electrochemically oxidized from its reduced basal state, the amine-benzenoid structures of the polymer backbone are converted into quinoids, accompanied by the release of protons. This process activates at a milder potential compared to water splitting.^6,7^ Sample acidification was confirmed by monitoring the pH via a potentiometric sensor located in front to the PANI proton pump. Optical pH sensing of proton release is indeed also possible, as demonstrated for the water splitting process at carbon electrodes.^8^ While not shown yet, the combination of an electrochemical actuator for proton release with a planar optode sensor, as those traditionally used for chemical imaging,^9,10^ is expected to provide valuable spatially and temporally resolved analysis.

In such a direction, this letter reports on the investigation of 2D acidification of samples at environmental and physiological pH (ca., 7.0) by electrochemically activated PANI deposited on a gold mesh. The analytical tool to demonstrate the concept was chemical imaging by means of a planar pH optode, as illustrated in Figure 1a. An optode–PANI sensor–actuator system in where the sample is sandwiched between the two elements (500 μm gap) was developed and utilized. The PANI was electropolymerized on the surface of a 4 μm thick gold mesh (open area of 70%) through cyclic voltammetry (200 scans from −0.35 to 0.85 V at 100 mV s^–1^ in 0.1 M aniline/0.5 M H_2_SO_4_ solution), providing a newly developed proton source (Figures S1 and S2). The pH optode (2.5 cm diameter) was based on either the HPTS derivative (1-hydroxypyrene-3,6,8-tris-bis(2-ethylhexyl)-sulfonamide)^11^ or EE (ethyl eosin) as the indicator dyes (with the corresponding reference dye). The optode readouts are based on a ratiometric approach between the fluorescence intensities of the corresponding indicator and reference dyes, which changes with pH. Details are provided in the Supporting Information (Table S1 and Figure S3).

**Figure 1:**
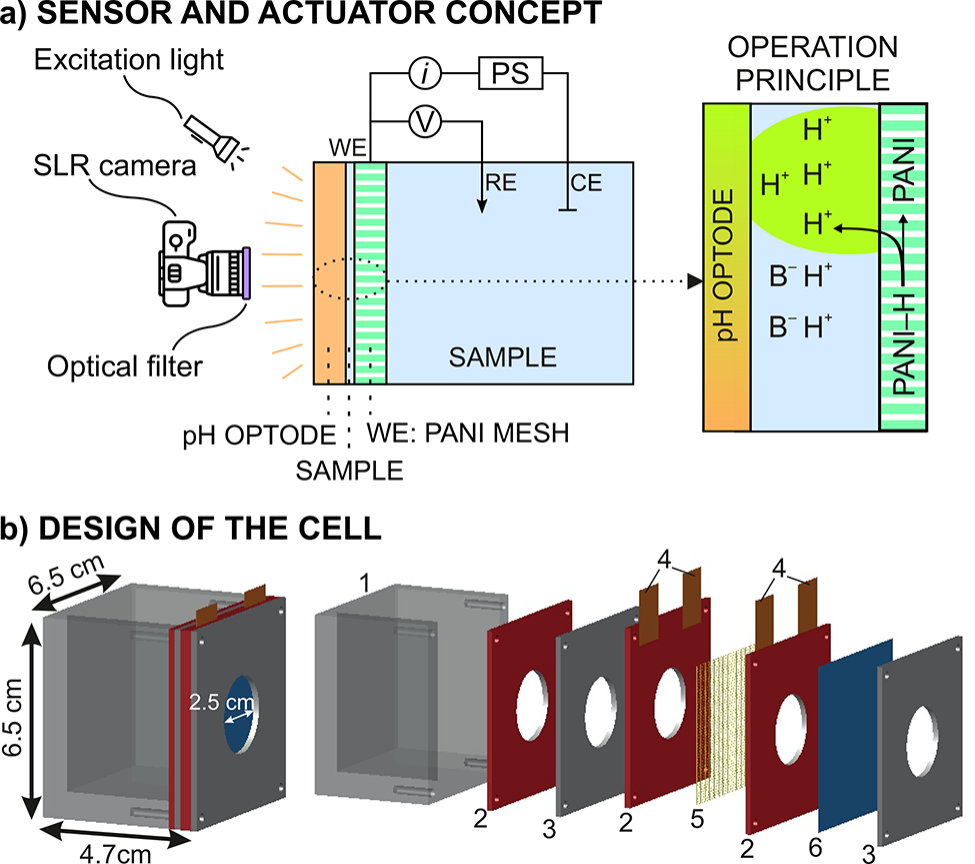
(a) The sensor and actuator concept with a magnification of the area (optode–sample–PANI) where the acid–base titration happens. WE, working electrode; RE, reference electrode; CE, counter electrode; PS, power supply (potentiostat); B^–^, deprotonated base. (b) Design of the cell conceived to perform the experiments. (1) Container to host the bulk solution, RE, and CE; (2) 0.50 mm thick rubber; (3) frame to provide rigidity; (4) electrical connections to (5) the PANI-gold-mesh and (6) pH optode.

The optode–sample–PANI structure was in turn in contact with the bulk sample (Figure 1a), which immediately passes through the PANI mesh to the narrow compartment between the optode and mesh while the solution is added to the bulk cell compartment. The counter and reference electrodes were placed in the bulk solution, with the PANI mesh being the working electrode of the three-electrode cell, which is additionally connected to the potentiostat. The optical readout was triggered by a light pulse to excite the dyes (indicator and reference) in the optode (405 nm UV LED for the HPTS and 470 nm blue LED for the EE), and the emitted fluorescence was collected using an SLR-camera focused on the planar pH optode. For more specific details, the reader is referred to the Supporting Information.

Initially, the PANI was in its reduced state, or protonated form (labeled as PANI-H), and the pH of the sample (dictated by the buffering species present in it) was read by the optode in the form of a 2D image. Then, upon electrochemical activation of PANI, a flux of protons was released from the PANI film to the sample.^6^ This proton flux shifted the acid–base equilibrium of any base species (prone to be protonated) in the sample close to the PANI mesh (represented as B^–^ in Figure 1a). Once the base species were consumed/protonated, the rest of the protons released from the PANI can rapidly diffuse along the sample thickness, therefore producing its acidification. As a result, a change in the optical readout was expected. Figure 1b presents the design of the experimental cell developed to demonstrate the 2D acidification concept, a real picture of which is provided in Figure S4.

First, we investigated the acidification of solutions comprising different buffer concentrations (1–20 mM phosphate, pH 7.0 measured with the pH-meter). The proton release from the PANI mesh was activated by applying a potential equal to the open-circuit potential (OCP) plus 0.4 V (applied with respect to the Ag/AgCl reference electrode) for 180 s.^7^ Simultaneously, images were acquired with either the HPTS or EE optodes, with a higher time resolution at the beginning of the applied pulse. While the HPTS optode is known to be fairly accurate covering the pH range from 8.5 to 4.5 (p*K*_a_^HPTS^ = 6.9), the EE optode was used to detect pH from 4.5 to 1.1 (p*K*_a_^EE^ = 2.3); see Figure S3 in the Supporting Information for the calibration graphs of the optodes. Figure 2 shows representative 2D images of a selected region of interest (ROI) of the optodes before, during, and after the 180 s pulse. The entire images provided by the optodes, together with the location of the ROI, which was selected from an optode area presenting no initial inhomogeneities, are provided in Figure S5.

**Figure 2:**
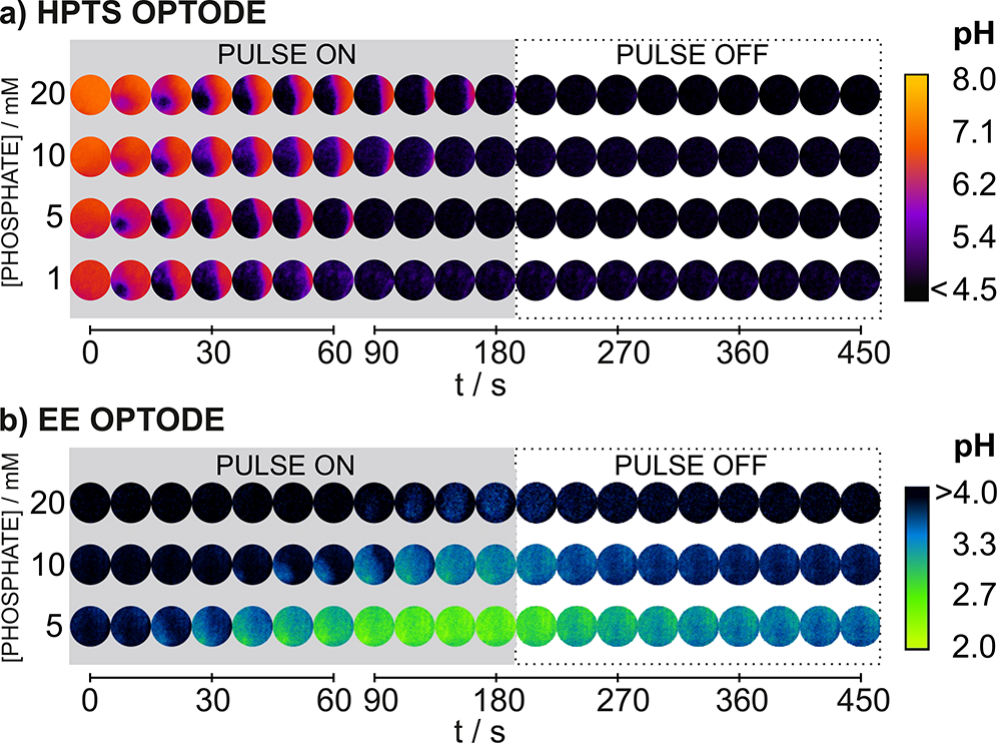
False color images of ROIs for (a) the HPTS-based optode and (b) EE-based optode responses before, during, and after the electrochemically modulated proton release from PANI (0.4 V + the OCP versus the Ag/AgCl RE for 180 s). The gray area represents the duration of the pulse.

Scales of “false” color are illustrated for each optode. Notably, optode images were not entirely homogeneous, which was especially remarkable in the entire images but not in the ROI ones. This is likely due to some residues from the used solutions (buffer samples and/or the H_2_SO_4_ in the PANI regeneration step) that stayed in the optode surface (initial images of the optode before applying the activation potential already revealed some inhomogeneities, Figure S5). Using more rigid materials for the mesh and optode substrates would better define the thin-layer sample confinement and facilitate more effective rinsing between samples, which will be considered in further investigations. Overall, the analysis of the ROIs permits minimizing such inhomogeneities while aiming for evidence of PANI-based acidification. Nevertheless, the use of the entire optode area will be necessary in future analytical applications targeting spatial resolution of the analyte concentration.

Prior to stepping the potential (i.e., 0 s), the optode response represents the initial (original) pH of each sample. The HPTS optode read a pH ranging from 6.5 to 6.8 (Figure 2a), values that are slightly lower than those measured with the pH meter (7.0 ± 0.1). Discrepancies between the pH meter and the optode have been commonly explained by a change in the ionic strength of the sample, which may influence the surface potential of the optode itself.^12^ In contrast, the EE optode read initial “fictitious” values close to pH 4.0, because the initial sample pH is higher than the upper limit of detection in the dynamic range of response (Figure S3). Also, we tested only the three solutions with the higher phosphate concentrations (20, 10, and 5 mM) with the EE optode to avoid inaccurate results caused by the dye leaching (0.95% per hour, Figure S6). Based on these results, the use of this optode for significantly long periods is not advisible.

Inspecting first the HPTS optode response once the potential pulse is on, the (false) color (and so the pH) was found to change in the entire range of response of the optode within the 180 s of the potential application for all the tested solutions. The lower the phosphate concentration in the sample, the sooner the total color change appeared in the ROI (ca. 60, 90, 150, and 180 s in 1, 5, 10, and 20 mM). The final pH achieved in the sample was found to be always <4.5. However, a quantification of the lowest attainable pH was not possible with the HPTS optode (pH of 4.5 is the lower limit of detection), and therefore, we performed complementary experiments with the EE optode.

The images provided by the EE optode (Figure 2b) revealed a “fictitious” initial pH of ca. 4 and a change in the (false) color of the entire ROI that is different for each solution at 180 s: the final pH reached in the sample increased with the phosphate buffer concentration and thus with increasing buffer capacity. After the pulse was switched off, there was a trend of increasing pH, which again was different for each sample. In essence, B^–^ species diffuse from the bulk sample solution to the thin compartment formed in between the optode and the PANI through the pores of the mesh, causing a gradual increase in the pH in the absence of any proton release. Without holding the PANI activation, the pH in the sample tended to return to the original value, which will occur sooner for higher phosphate concentrations. Ideally, the maintenance of the polarization potential would allow keeping the achieved acidification, and hence, it would be possible to dynamically monitor concentration changes utilizing an optode for another analyte (requiring acidification for its detection) rather than pH.

Upon PANI activation, most of the proton release takes place within the initial 2 min and independently of the phosphate concentration in the sample, according to overlapping chronoamperometric response recorded for the PANI mesh (Figure S7). Effectively, the charge calculated under the current curve was rather constant for all the tested conditions (0.6821 ± 0.0631 C), confirming the excellent reproducibility of the proton release from the PANI mesh after appropriate regeneration between measurements (0 V versus the reference electrode, 180 s, 10 mM H_2_SO_4_). 90% of the total charge was reached after the first 122 ± 7 s, meaning that most of the pH change in the sample is expected to occur in that period. While the same number of protons is always delivered from the PANI to the solution, this is differently employed in breaking off the different buffer capacities of the samples. The higher the phosphate concentration, the higher the buffer capacity, and hence, the higher proton charge is needed to overcome it, resulting in fewer protons to acidify the sample. Accordingly, a lower pH is expected to be reached for the 5 mM phosphate sample compared with the 20 mM sample, which is indeed what we observed with the EE optode (Figure 2b).

Figure 3a displays the pH quantification in a combined way for the HPTS and EE optodes (the corresponding individual plots are shown in Figure S8), considering the averaged pH measured in the entire ROI (i.e., from the data presented in Figure 2). In essence, the plot joins the HPTS optode readout from 0 s until the point that a constant pH value of 4.5 (the lower limit of detection) was displayed, together with the EE optode readout, from the point at which the pH starts varying from 4.0 (the upper limit of detection) until the end of the pulse and after. As observed, while the initial time for the pH decrease to be visualized did not dramatically differ between the tested samples (ca. 10 s), the higher the phosphate concentration, the higher the final pH value that was reached after 180 s (pH of 2.3, 2.6, and 3.2 for 5, 10, and 20 mM phosphate concentrations, respectively) and the more pronounced was the diffusion effect of B^–^ species (as above-described) after the polarization pulse stops (final pH of 3.1, 3.4, and 4–4.5, respectively). Also, it was evident that the minimum pH achieved in all the samples coincided with the maximum charge released from the PANI mesh (final flat zones of the charge curves in Figure 3b). A pH below 4.0 could be maintained in all the samples during ca. 200 s after the potential pulse was switched off.

**Figure 3:**
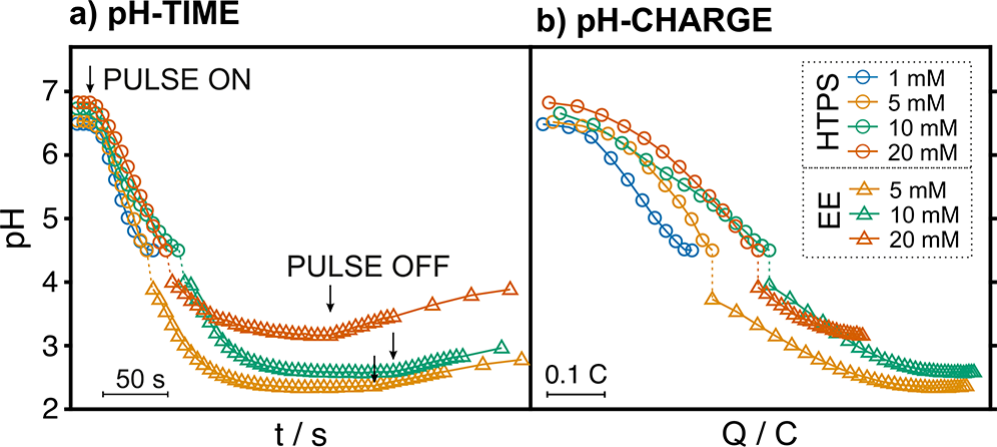
pH response of the HPTS and EE optodes in phosphate buffer solutions of different concentrations (100 mM NaCl) plotted in a combined way versus (a) time and (b) charge.

The demonstrated acidification levels are in principle suitable to further implement the sensor–actuator concept for combined sample pretreatment and analysis in such demanding applications as alkalinity (formal pH of 4.0, pH or CO_2_ optode)^7^ and dissolved inorganic carbon (pH 4.0 for >99% of conversion, CO_2_ optode) detections in the environmental field,^13^ whereas the determination of biomarkers or drugs such as penicillin (pH 4–4.5, other detector), certain immunoassays, and total sulfide in fluids and tissues (pH 4.8 for >99% of conversion, H_2_S optode) are important in the clinical domain,^14−16^ among others. In any case, the PANI mesh could be optimized in terms of porosity and the amount of deposited PANI to fine-tune the delivered charge of protons according to the application needs. Overall, the developed PANI mesh presents versatility to cover a wide range of buffer capacities in the sample, which will primarily affect the final pH that is obtained for a given charge of protons. Then, it is possible to replace the pH optode with another one to spatially follow the pertinent analyte after sample acidification. Moreover, the implementation of other analytical tools, such as electrochemical sensors, would be also accessible for discrete measurements rather than imaging.

To further illustrate the capability of the optode–PANI sensor–actuator system for obtaining 2D resolution in the sample acidification process, and to identify hence local differences in buffer capacity in the same sample, the experimental cell was modified with a spacer material that divided the main reservoir into two compartments (Figure 4a). Notably, the part of the spacer in contact with the PANI mesh was made of filter paper to allow electrical connection between the CE, RE, and the two solutions placed in each compartment (Figure S9).

Two buffer solutions of different phosphate concentrations (1 and 100 mM) were placed in each of the two compartments (Figure 4a). Then, the PANI was activated for 180 s for acidification, and pH images were dynamically obtained (HPTS optode). While the optode image did not show any remarkable spatial variance with the naked eye before acidification, two regions with rather different pH (ca. 6.7 and <4.5) were clearly distinguished after the acidification process (Figure 4a). The part of the image corresponding to the 1 mM phosphate solution displayed a substantial change in pH (dark purple part), while the 100 mM phosphate solution presented only a moderate change (orange part), as the proton release was not able to locally surpass a very high buffer capacity at the established experimental conditions. To the best of our knowledge, this experiment constitutes the very first 2D visualization of buffer capacity gradients within the same sample.

**Figure 4:**
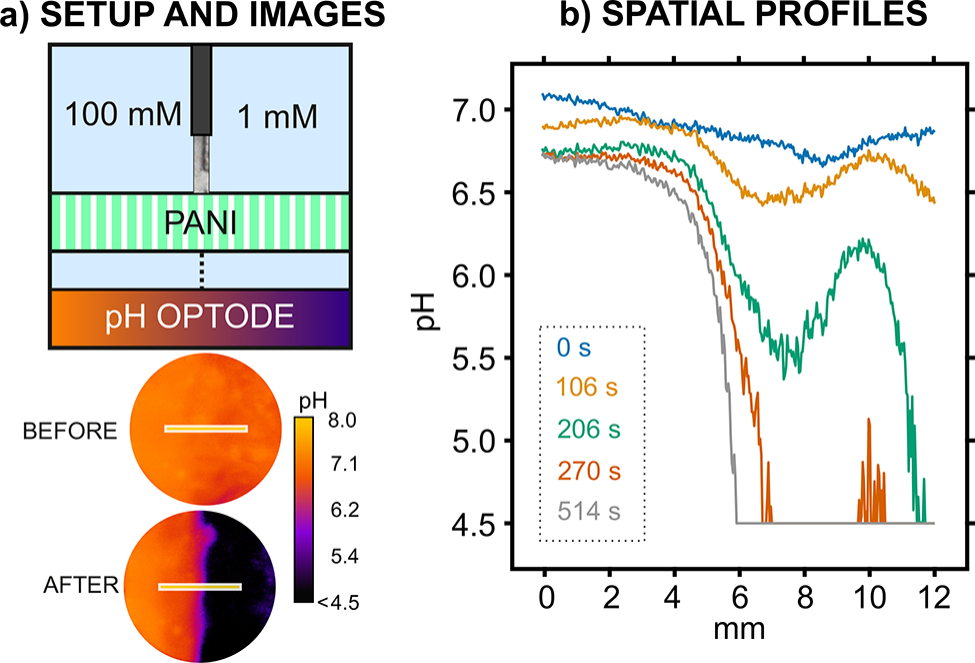
(a) Top: Scheme of the experimental setup for the two-solution experiment (1 mM and 100 mM phosphate buffer concentration in 100 mM NaCl). Bottom: Images of the HPTS before and after acidification. The yellow line indicates the selected linear spatial profile. (b) Plot of the pH spatial profiles observed at representative times of acidification.

All the sets of images were further analyzed to obtain the linear spatial variation of pH at every acquisition time. The total length of the analyzed line profile was 12 mm and was positioned in the middle of the optode image, so the separation of the two solutions occurs at approximately 6 mm. Some representative curves are depicted in Figure 4b. Before activation of the proton release (0 s), the pH readout can be regarded as constant over the inspected space (6.9 ± 0.1). Once the polarization starts, a change in pH could be observed along the line-profile, with the largest changes appearing in the region corresponding to the 1 mM phosphate solution (right part of the figure, >6 mm). The longer the time, the larger the pH change compared to the initial one: while the total change at 0 mm (corresponding to the 100 mM phosphate solution) was of ca. 0.4 pH units, the change at 12 mm was of more than 2.5 pH units. Then, there was a region at roughly 6 ± 1 mm where the two solutions were partially mixed. This was very evident in the curve obtained at 514 s (gray curve), where there is an initial constant region from 0 to 4 mm of pH 6.7 ± 0.1 and then a gradual decrease down to pH < 4.5 from 6 mm.

Overall, the developed sensor–actuator system has demonstrated 2D acidification down to pH levels that have been claimed as useful for many different applications (e.g., 4.0 for alkalinity and drug detection, 4.8 for total sulfide). The beauty of the concept relies also in the possibility to further exchange the sensor by another optode or electrochemical sensor to monitor the concentration of different analytes (rather than pH). Planar optodes, in particular, will offer high spatial and temporal resolution in two dimensions via imaging. Notably, it would be convenient to inspect the entire optode image rather than the ROI for better accuracy when further exploiting the developed sensor–actuator system. This will serve to study the heterogeneous distribution of pH-dependent analytes in complex biological systems with 2D resolution.

## Supporting Information Available

Experimental Section: electrochemical procedures, data treatment, optode compositions, experimental setups, additional images, and complementary experiments (PDF)
