# Supplementary material for: Imaging Sample Acidification Triggered by Electrochemically Activated Polyaniline
Source: Anal Chem. 2022 Sep 27;94(40):13647–51. doi: 10.1021/acs.analchem.2c03409 (PMC9558083; doi:10.1021/acs.analchem.2c03409)
Supplement: Supplementary file 1 — ac2c03409_si_001.pdf [file ac2c03409_si_001.pdf]

Supporting Information for:

Imaging Sample Acidification Triggered by Electrochemically Activated Polyaniline

Fabian Steininger[1†], Alexander Wiorek[2†], Gaston A. Crespo[2], Klaus Koren[1*] and Maria Cuartero[2*]

[1]*Aarhus University Centre for Water Technology, Department of Biology, Section for Microbiology, Aarhus University, 8000 Aarhus, Denmark*

[2]*Department of Chemistry, School of Engineering Science in Chemistry, Biochemistry and Health, Royal Institute of Technology, KTH, SE-100 44 Stockholm, Sweden*

Corresponding author (*): klaus.koren@bio.au.dk; mariacb@kth.se

Table of Contents

1. **Experimental Section** SI-2

Reagents, materials and instrumentation SI-2

Fabrication of the experimental cell. SI-2

Electropolymerization of PANI on the gold mesh SI-3

Optodes fabrication. SI-3

Optical measurements and data treatment SI-3

Procedure for proton release, rinsing- and regeneration step with the PANI-Au mesh. SI-4

2. **Tables** SI-5

Table S1 .... SI-5

3. **Figures** SI-6

Figure S1 .... SI-6

Figure S2 .... SI-7

Figure S3 .... SI-8

Figure S4 .... SI-9

Figure S5. .... SI-10

Figure S6 .... SI-11

Figure S7 .... SI-12

Figure S8 .... SI-13

Figure S9 .... SI-14

References SI-15

1. Experimental Section

Reagents, materials and instrumentation. Aniline (ACS reagent grade, ≥99.5%), sulfuric acid (99% w/w), potassium phosphate monobasic (ACS reagent grade, ≥99%), sodium chloride (99.5%), sodium hydroxide (ACS reagent, ≥97%), sodium citrate tribasic dihydrate (ACS reagent, ≥99%), tetrahydrofuran (anhydrous, ≥99.9%), ethyl eosin and perylene (≥99%) were purchased from Sigma Aldrich. Di-potassium hydrogen phosphate (99%) and Hydrochloric acid (reagent grade, 1 M) were purchased from VWR. Macrolex® fluorescence yellow 10GN was obtained from Lanxess, Germany. Hydromed D4 was purchased from AdvanSource Biomaterials. Monocrystalline diamond powder was purchased from Microdiamant AG, Switzerland, and carbon black was purchased from Kremer Pigmente, Germany. The lipophilic pH indicator HPTS (1-Hydroxypyrene-3,6,8-tris-bis(2-ethylhexyl)-sulfonamide was provided by Dr. Sergey Borisov, Graz University of Technology, Austria). The gold mesh (0.004 mm thickness, 333 wires/inch, 70% open area, 50x50 mm) electrode was purchased from Good-Fellow, Germany. The platinum rod counter electrodes (Model 6.0331.000, Metrohm Nordic AB) and the single junction Ag/AgCl/3 M KCl reference electrode (Model 6.0726.100, Metrohm Nordic AB) were purchased from Metrohm Nordic AB. All solutions were prepared with ultrapure water having resistance of 18.2 MΩ cm (Milli-Q water systems, Merck Millipore).

Electrochemical experiments were performed using a PGSTAT204 Autolab potentiostat (Metrohm Nordic AB) connected to a PC and operated using the Nova 2.1.5 software. A pH-meter (914 pH/Conductometer, Metrohm) was used for additional pH-measurements (6.0228.000, Metrohm). The imaging of the HPTS-based optodes was achieved with a SLR camera (EOS 1300D, Canon, Japan) with a macro-objective lens (Macro 100 F2.8 D, Tokina, Japan), a yellow 455 nm long-pass filter (GG455 SCHOTT, 52 mm × 2 mm) with another plastic filter (#10 medium yellow; LEEfilters.com) attached in front of the long-pass filter to minimize background fluorescence. Excitation of the optode was accomplished using a 405 nm UV LED (r-s components, Copenhagen, Denmark). The LED was controlled by a USB-controlled LED driver unit (trigger box, imaging.fish-n-chips-de). For collection of images, the SLR camera and LED were controlled using the software look@RGB (imaging.fish-n-chips-de). For imaging the EE-based optodes, the same setup was used but using an orange 530 nm longpass filter and a blue (470 nm) LED (r-s components, Copenhagen) instead of the filter and excitation light mentioned above. Microscope pictures were taken using a home-built Cerna mini microscope (parts purchased from Thorlabs) using a 5MP camera (CS505CU, Thorlabs).

Fabrication of the experimental cell. The cell-parts were designed in AutoCAD 2022 and fabricated using a co-polyester filament (CPE+, Ultimaker B.V., Netherlands) using a 3D printer (Model Ultimaker 3, Ultimaker B.V., Netherlands), see **Figure 1b** in the main manuscript and **Figure S1b**, **S4** and **S9** in the *Supporting Information*. The cell was then assembled using two 40kg pressure one-hand bar clamps (Cocraft, Claes Ohlson) to seal the cell, with screws for alignment of the cell parts. For electropolymerization of the PANI, the cell (**Figure S1b**) constituted a main liquid container (1) to host the monomer solution (0.1 M aniline, 0.5 M sulfuric acid) and the counter and reference electrodes. Then, the working electrode (5) (gold mesh) was tightened and stretched as much as possible without causing damage to it, between two rubbers (2) and two 3D-printed frames (3) to maintain rigidity and provide a circular contact area between liquid and optode with a 2.5 cm diameter. Electrical connections to the mesh were achieved by attaching four pieces of copper tape (4) to the rubbers, putting them in contact with the mesh. Then, to provide a good mass transport of the monomer to the mesh-surface, a back-container (6) was attached to complete the cell, allowing diffusion of monomer from both sides of the mesh.

The cell comprising the optode (i.e., the actuator-sensor system, **Figure 1b**, **Figure S4** and **Figure S9**) was designed in the same way as for the PANI electropolymerization but replacing the back-container (6 in **Figure S1b**) with the optode (6 in **Figure 1b** in the main manuscript) and adding another stabilizing-frame (3 in **Figure 1b** in the main manuscript).

Electropolymerization of PANI on the gold mesh. The electro polymerization of PANI followed the same procedure as described elsewhere,[1] with minor modifications. Initially, the open circuit potential (OCP) was registered for 10 s, confirming that electrical connections were adequate. Then, the potential was held at 1.3 V for 10 s to initiate nucleation of PANI on the gold surface, whereafter the potential was swept between –0.35 and 0.85 V for 200 scans at 100 mV/s, under moderate stirring (150 rpm), which was started after the 10th scan, with the resulting CVs presented in **Figure S2**. The stirring was not started until after the 10th scan was completed, because convection would mechanically detach newly formed PANI from the bare gold surface. After 10 scans, enough PANI was formed on the surface to allow a robust attachment to the electrode without the risk from stirring mechanically detaching the PANI. These parameters were used for all experiments presented herein.

The CVs in **Figure S2a** presents selected cycles during the first 50 scans, exhibiting three distinct oxidation peaks (216 mV, 560 mV and 838 mV in the 10th scan) corresponding to different electrochemical processes in the PANI-film.[2, 3] The first peak (216 mV) is assigned to the oxidation of the fully reduced form, leucoemeraldine, into the partially oxidized form emeraldine, and the third peak (838 mV) is further assigned to the transition from emeraldine into the fully oxidized pernigraniline.[2, 3] The second peak has previously been assigned to degradation processes in the Polyaniline film, mainly as result from hydrolysis of imine bonds in the emeraldine backbone.[4, 5] The electropolymerization of PANI resembles that of our previous work,[1] indicating that hydrolysis occurs during the early stages of film growth, whereafter it is no longer visible after the 40th scan, thus avoiding loss of conductivity of the material.[4] Furthermore, a shift of the peaks can be observed with increasing number of scans (**Figure S2b**), explained by an increase in resistance with increasing thickness of the PANI-film.[3]

Optodes fabrication. The optodes were prepared by knife coating a sensor cocktail (see **Table S1**) onto a transparent, dust-free polyethylene terephthalate (PET) foil (Puetz Folien, Germany) using a film applicator (Byk-Gardner GmbH). To minimize the impact of background light and influence of the color change of PANI during oxidation (proton release), an optical isolation layer was knife coated on top of the sensing layer, resulting in a total film thickness of ~10 µm.

Optical measurements and data treatment. All optical measurements were performed in a dark chamber to minimize the influence of background light. The optodes were fitted into the measuring cell and calibrations were performed as follows: for the HPTS based optode images were recorded in 10 mM phosphate buffer solutions (pH 2.5, 4, 4.5, 5, 5.5, 6, 6.5, 7, 7.5, 8, 8.5, 9, 11.5). For the ethyl eosin based optode images were recorded in 10 mM citrate buffer solutions (pH 1.2, 1.5, 2, 2.5, 3, 3.5, 4, 4.5, 5). All calibration solutions contained 100 mM NaCl as the background electrolyte. Images were taken in 1-minute intervals for a total of 5 minutes at each pH-value to ensure that a steady-state signal was reached.

Collected images were analyzed following an intensity-based ratiometric approach using the software ImageJ (imagej.nih.gov/ij/).[6] The images were split into their respective color channels (red, blue and two green) by the *look@RGB* software and the ratiometric signal was calculated from the ratio (utilizing the ImageJ plugin Ratio Plus) between the blue channel (emission of perylene) and the red channel (emission of HPTS) for the HPTS optode. For the EE optode the ratio between the green channel (emission of Macrolex® fluorescent yellow

10GN) and the red channel (ethyl eosin) was calculated. The resulting ratios were plotted versus the pH values of the calibration solutions and the datapoints were fitted using a Sigmoidal fit (**Figure S3**). A Macro for ImageJ was used to convert the obtained ratios from imaging to pH values by simply inputting the fitting parameters of the calibration to the macro, according to Merl et. al.[7]

Procedure for proton release, rinsing- and regeneration step with the PANI-Au mesh. Before the PANI-mesh was introduced into either the sample solution or the regeneration solution (10 mM H_2SO_4, see below), it was rinsed by stirring deionized water in the same cell for 1 minute. This step was repeated two times to clean the PANI from acid- or phosphate buffer residues, before introducing the PANI film to a new solution and run the corresponding experiment. Proton releases were performed by applying 0.4 V versus the open circuit potential (OCP) for 180 s in the sample solution (phosphate buffer with 100 mM NaCl as background electrolyte), as described more in detail in the main manuscript. Between proton release experiments, the PANI film was introduced into 10 mM H_2SO_4, where it was stirred for 1 minute. Thereafter, the PANI film was subject to an applied potential of 0 V versus the reference electrode for 180 s. This step assured that the PANI was in its reduced state (emeraldine) and allowed for reproducible proton releases.[1, 2]

2. Tables

Table S1. Optode cocktail compositions. Listed components were mixed with a stock solution of 75 mg Hydromed D4 / 1.5 g THF (5 wt%) for the optode cocktail preparation. Percentage in brackets refer to wt% relative to Hydromed D4.

Optode	Optode Cocktail Composition mg/mL THF (wt%)	
HPTS-optode	HPTS (indicator dye)	1.5 (2)
	Perylene (reference dye)	1.5 (2)
	Diamond powder (Sensing layer only)	75 (100)
	Carbon black (Optical isolation layer only)	24 (32)
Ethyl Eosin-optode	EE (indicator dye)	1.5 (2)
	Macrolex® fluorescent yellow 10GN (reference dye)	1.5 (2)
	Diamond powder (Sensing layer only)	75 (100)
	Carbon black (Optical isolation layer only)	24 (32)

3. Figures

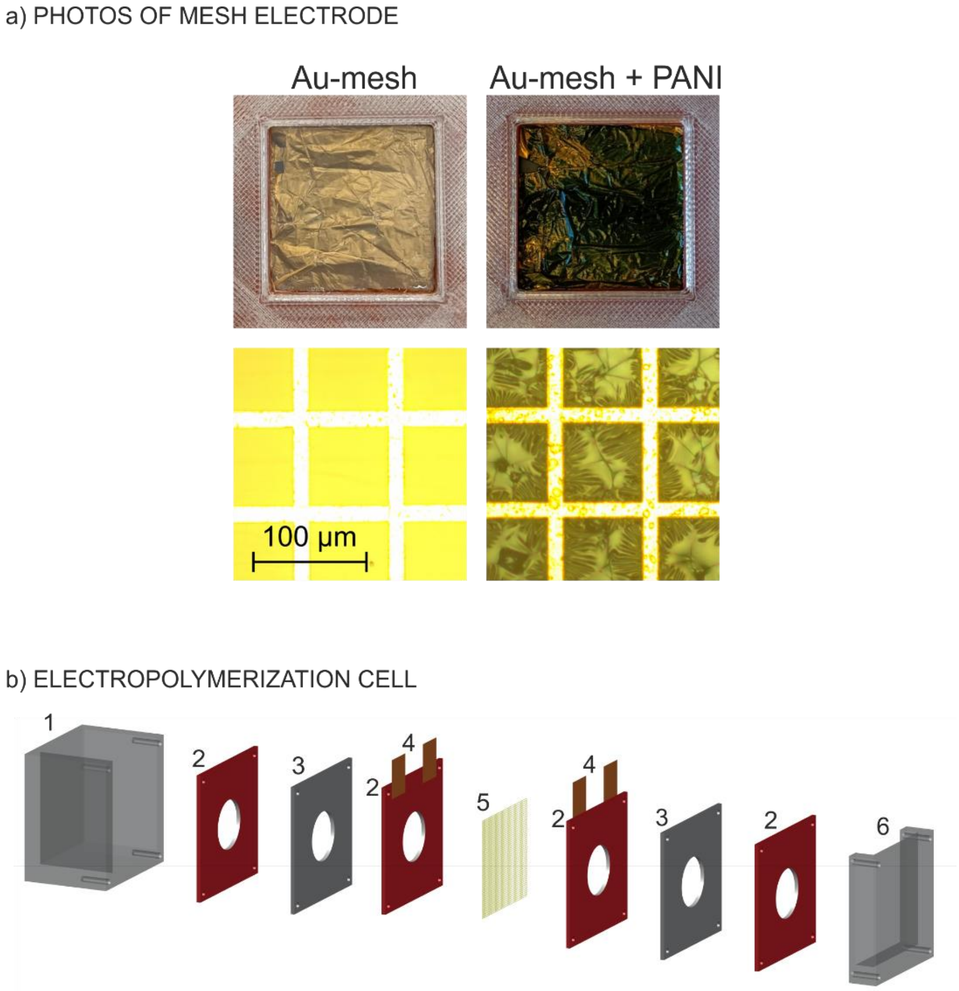

Figure S1. (a) Picture of Au-mesh with and without PANI inside a rectangular mesh and under the microscope. (b) Illustration of the electropolymerization cell used for PANI-synthesis. 1: container to host the bulk solution, RE and CE, 2: a 0.50 mm thick rubber, 3: a frame to provide rigidity, 4: copper connections, 5: the PANI-gold-mesh, and 6: the back container for the solution.

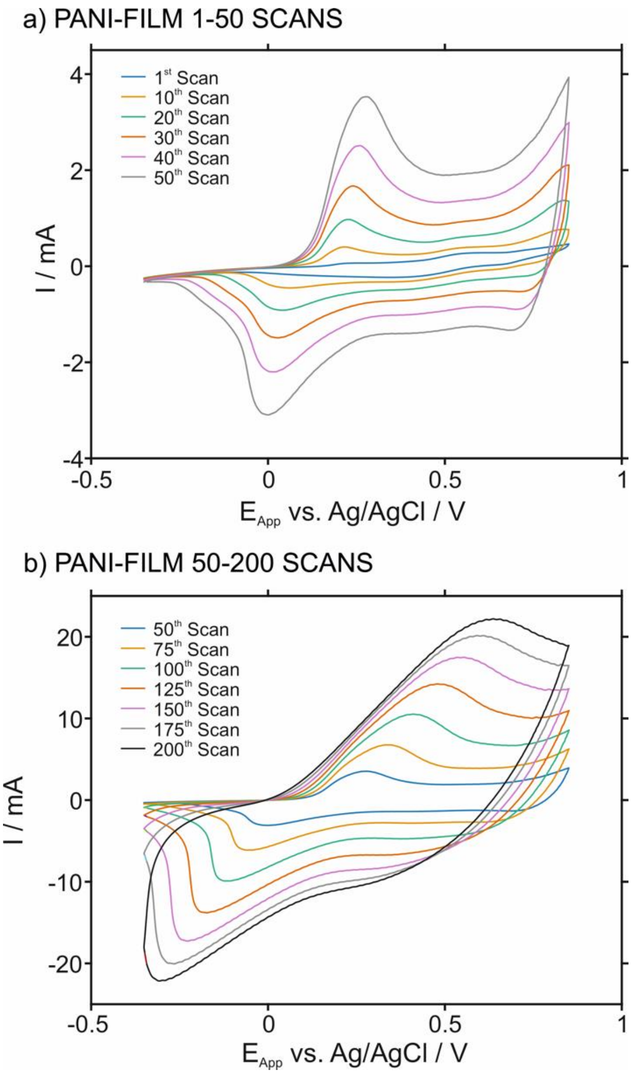

Figure S2. The cyclic voltammograms of selected scans from the PANI electropolymerization on the Au mesh (from –0.35 to 0.85 V vs Ag/AgCl reference, 100 mV s^{-1}, 0.1 M Aniline/0.5 M H_2SO_4), where **(a)** displays the trend over the 50 first cycles, and **(b)** displays the trend from the 50th to the 200th cycle.

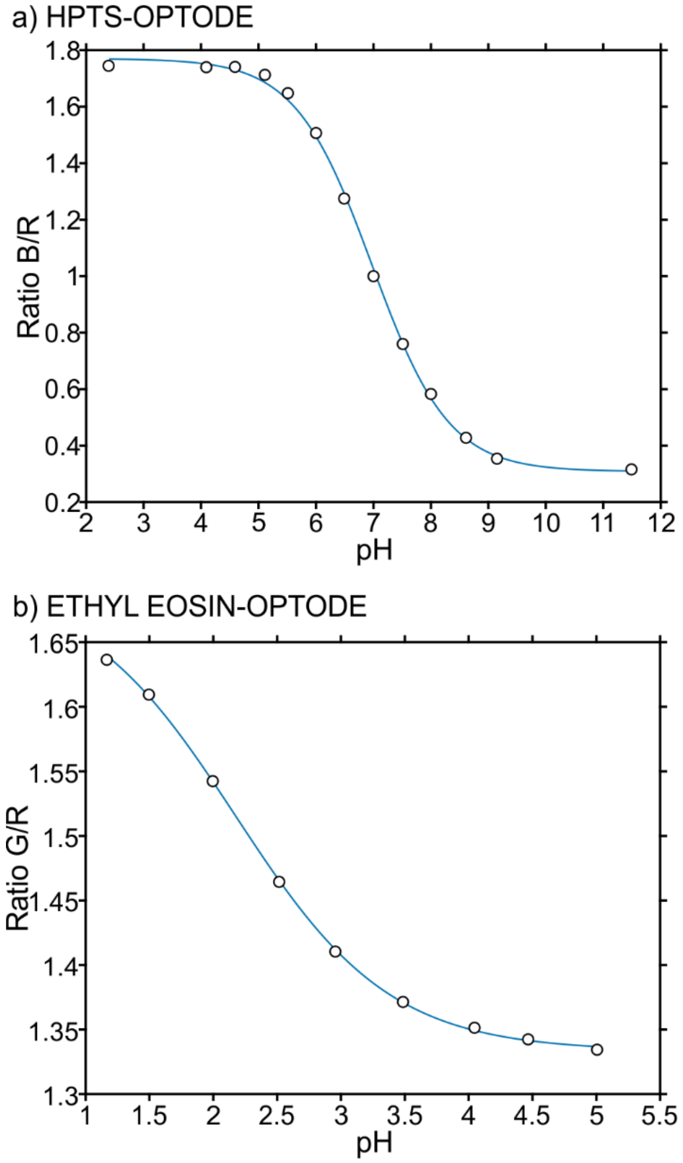

Figure S3. Calibrations of the optodes: **(a)** HPTS-based optode (background: 10 mM Phosphate buffer and 100 mM NaCl); and **(b)** EE-based optode (background: 10 mM citrate buffer and 100 mM NaCl).

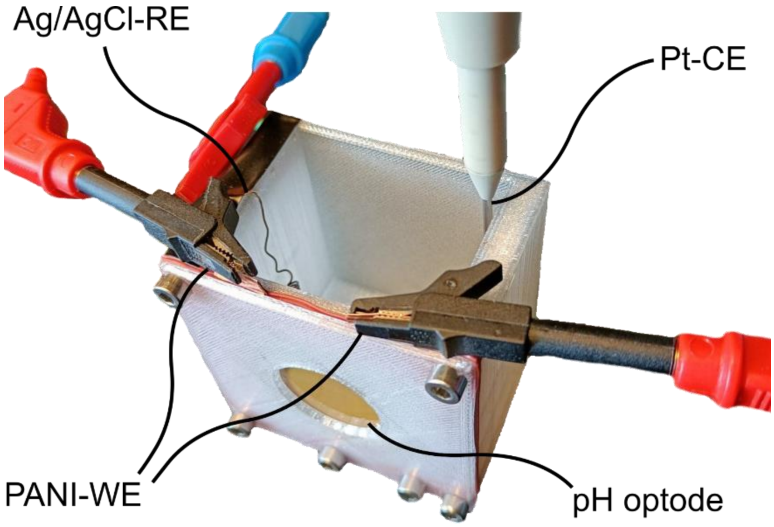

Figure S4. Picture of the cell with PANI-mesh, CE, RE and optode.

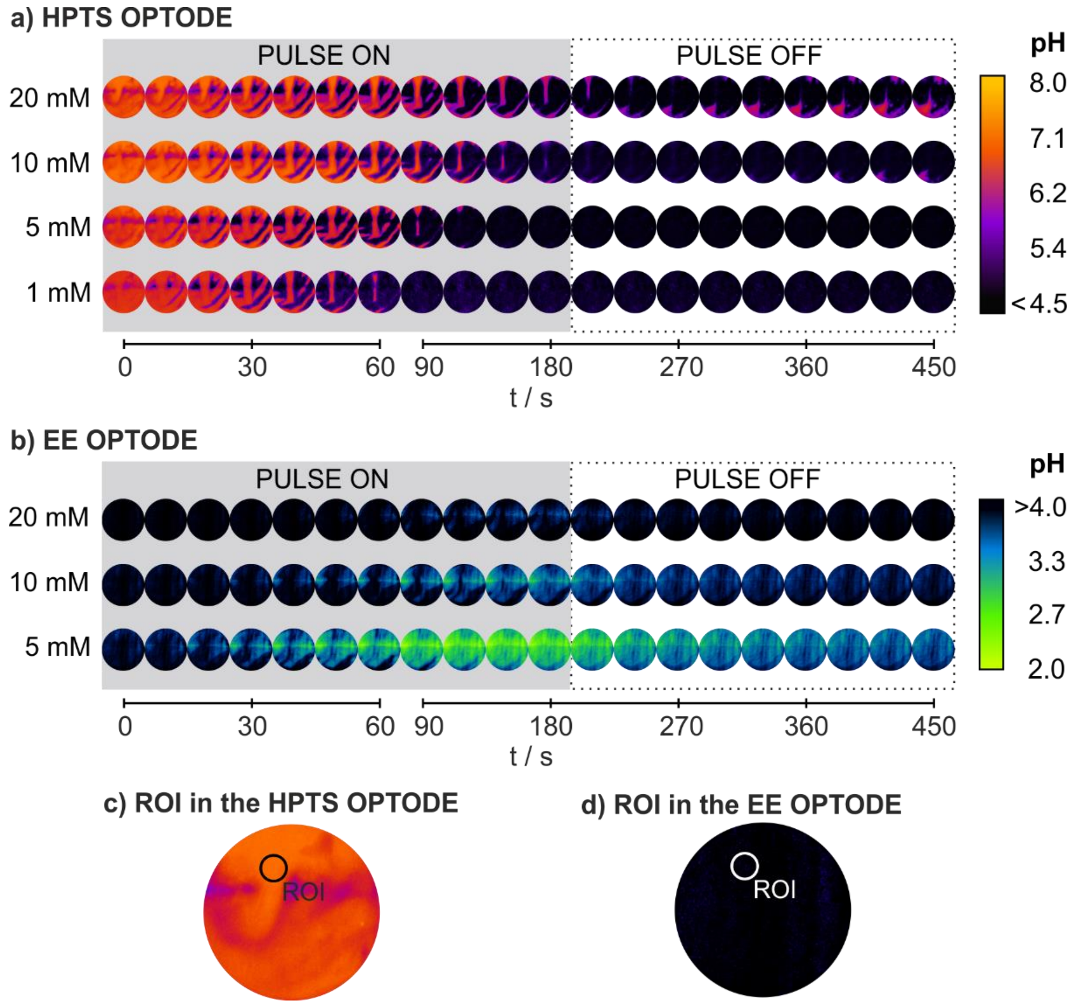

Figure S5. False color images of **(a)** the HPTS based optode and **(b)** the EE based optode response before, during and after the electrochemically modulated proton release from PANI (0.4 V vs OCP for 180 s). The gray area represents the duration of the pulse. The ROIs of figure 2 are further presented in **(c)** for the HPTS based optode, and **(d)** for the EE based optode. The optode areas were designed circular, with a diameter of 2.5 cm.

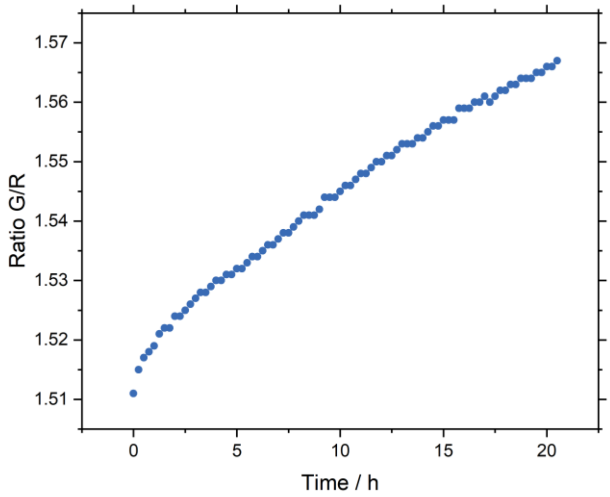

Figure S6. Leaching behavior of the EE optode in D4 matrix: plot of the G/R ratio in a stirred solution of 10 mM citrate buffer (pH=3, 100 mM NaCl background). The leaching rate was calculated to 0.95 % per hour.

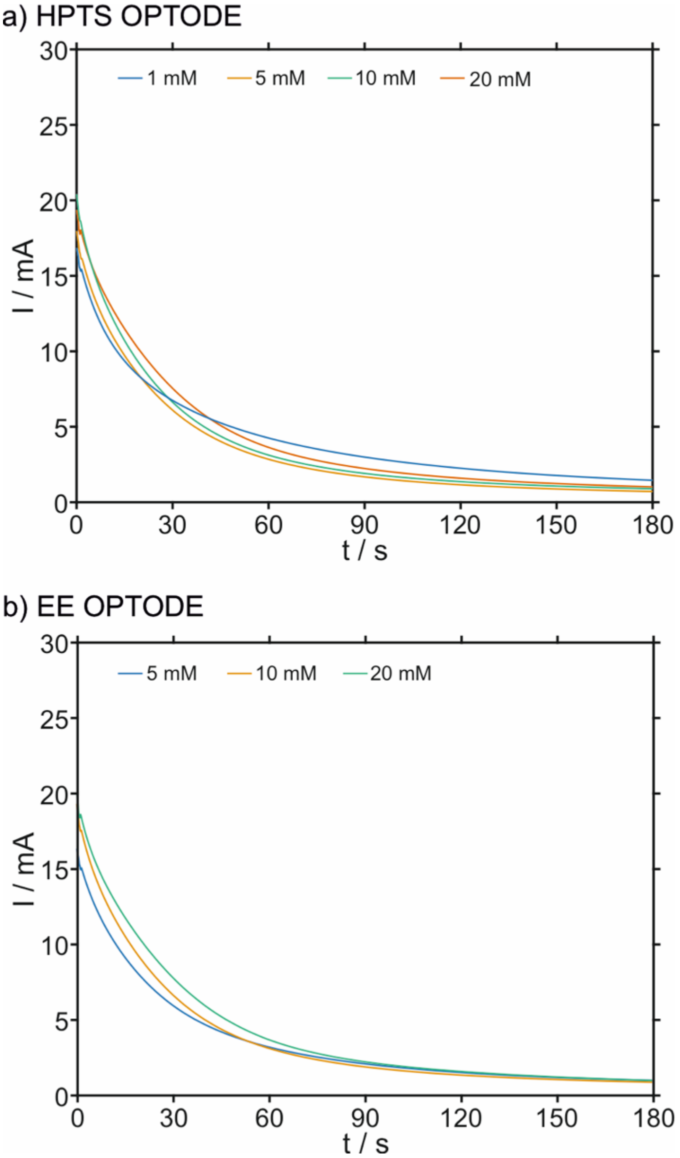

Figure S7. The chronoamperometric responses from the proton release (0.4 V vs OCP for 180 s) in solutions with different concentrations of phosphate buffer with **(a)** the HPTS optode and **(b)** the EE optode.

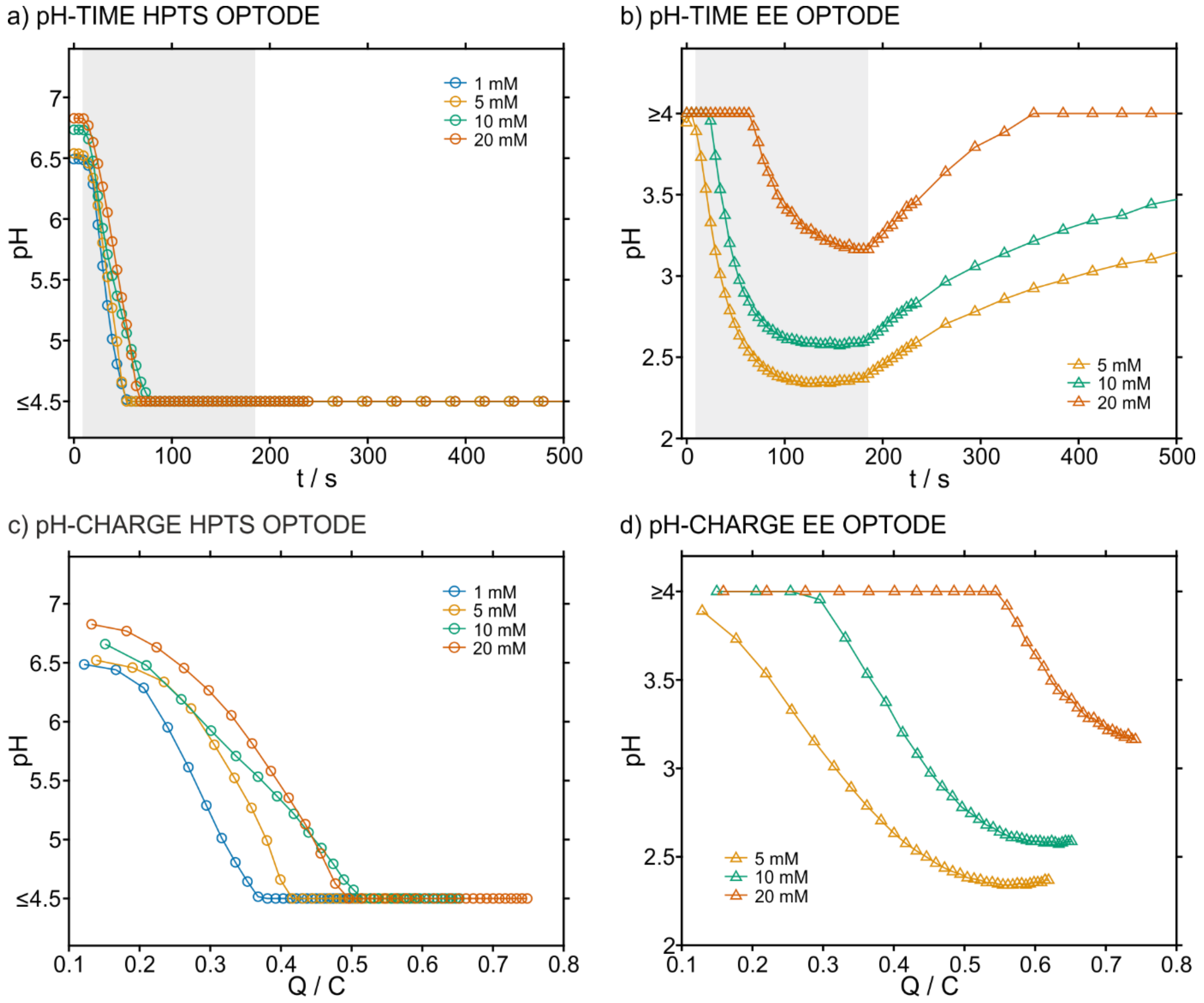

Figure S8. The pH-time profiles for **(a)** the HPTS optode and **(b)** the EE optode, with the corresponding pH-charge profiles, **(c)** and **(d)** respectively, in different concentrations of phosphate buffer solutions.

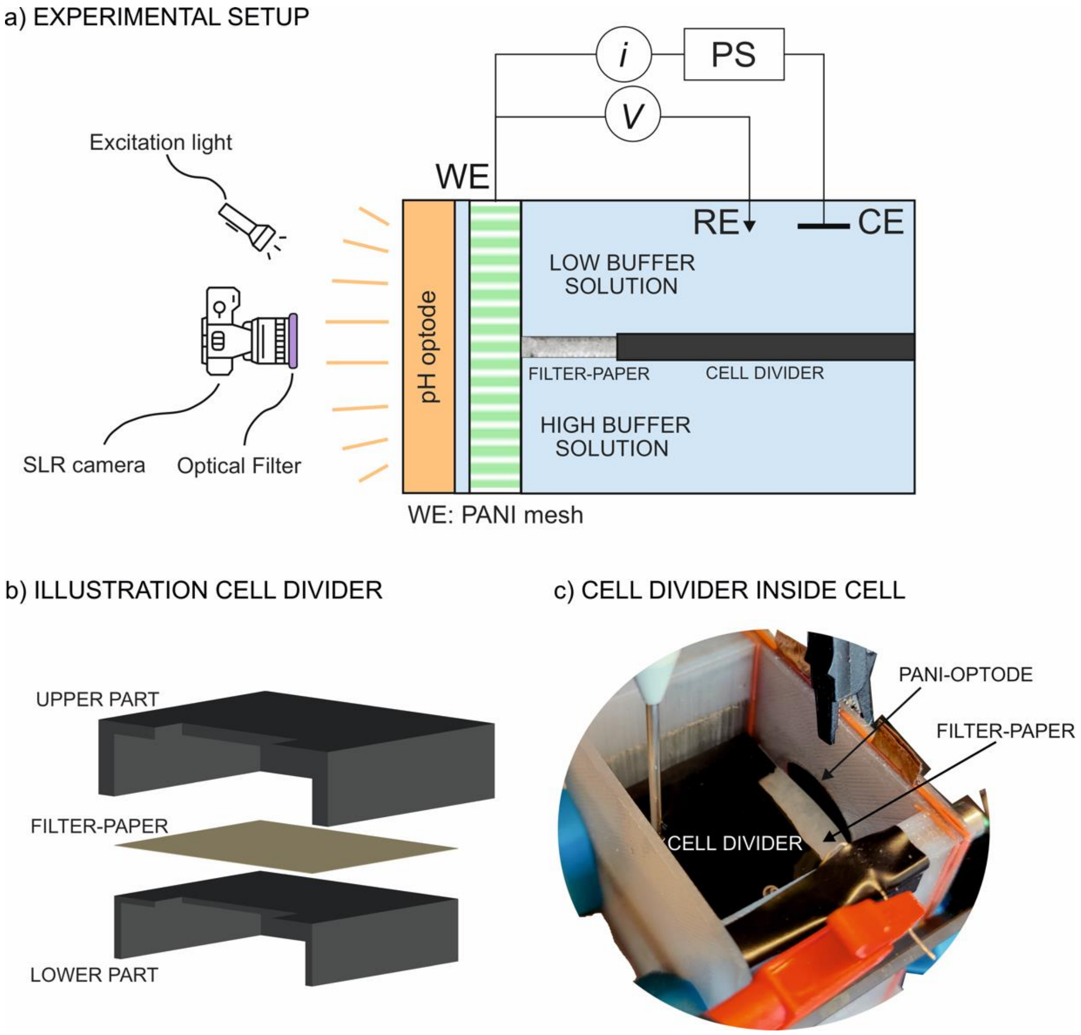

Figure S9. Experimental setup for the buffer-gradient experiment. **(a)** The image presents a sketch of the experimental configuration, where the cell-divider is a 3D-printed holder for a filter-paper, separating the upper and lower parts of the cell, hence allowing for each compartment to be filled with different solutions with minimal mixing. **(b)** Illustration of the cell divider parts. **(c)** Real picture of the setup. WE: working electrode, RE: reference electrode, CE: counter electrode, PS: power supply.

References

1. Wiorek, A.; Hussain, G.; Molina-Osorio, A.F.; Cuartero, M.; Crespo, G.A. , Reagentless Acid−Base Titration for Alkalinity Detection in Seawater. *Analytical Chemistry* **2021,** *93*, 14130−14137.
2. Wiorek, A.; Cuartero, M.; De Marco, R.; Crespo, G.A., Polyaniline Films as Electrochemical-Proton Pump for Acidification of Thin Layer Samples. *Analytical Chemistry* **2019,** *91*, 14951−14959.
3. Genies, E.M.; Boyle, A.; Lapkowski, M.; Tsintavis, C., Polyaniline: A historical survey. *Synthetic Metals* **1990,** *36*, 139-182.
4. Zhang, H.; Li, H.; Wang, J., Capacitance Fading Induced by Degradation of Polyaniline: Cyclic Voltammetry and SEM Study. *Advanced Materials Research* **2012**.
5. Chen, W.C.; Wen, T.C.; Gopalan, A., The inductive behavior derived from hydrolysis of polyaniline. *Electrochemica Acta* **2002,** *47* (26), 4195-4206.
6. Larsen, M.; Borisov, S.M.; Grunwald, B.; Klimant, I.; Glud, R.N., A simple and inexpensive high resolution color ratiometric planar optode imaging approach: application to oxygen and pH sensing. *LimnologY and Oceanography: Methods* **2011,** *9*, 348–360.
7. Merl, T.; Koren, K., Visualizing NH_3 emission and the local O_2 and pH microenvironment of soil upon manure application using optical sensors. *Environment International* **2020,** *144*, 106080.
